# Understanding gut-liver axis nitrogen metabolism in Fatty Liver Disease

**DOI:** 10.3389/fendo.2022.1058101

**Published:** 2022-12-15

**Authors:** Teresa C. Delgado, Javier de las Heras, María L. Martínez-Chantar

**Affiliations:** ^1^ Liver Disease Lab, Center for Cooperative Research in Biosciences (CIC bioGUNE), Basque Research and Technology Alliance (BRTA), Derio, Bizkaia, Spain; ^2^ Congenital Metabolic Disorders, Biocruces Bizkaia Health Research Institute, Barakaldo, Spain; ^3^ Division of Pediatric Metabolism, Department of Pediatrics, CIBERer, Cruces University Hospital, Barakaldo, Spain; ^4^ Department of Pediatrics, University of the Basque Country (UPV/EHU), Leioa, Spain; ^5^ Centro de Investigación Biomédica en Red de Enfermedades Hepáticas y Digestivas (CIBERehd), Carlos III National Health Institute, Madrid, Spain

**Keywords:** Gut-liver axis, nitrogen metabolism, Fatty Liver Disease, ammonia, glutamine, glutaminase, urea cycle

## Abstract

The homeostasis of the most important nitrogen-containing intermediates, ammonia and glutamine, is a tightly regulated process in which the gut-liver axis plays a central role. Several studies revealed that nitrogen metabolism is altered in Metabolic Dysfunction-Associated Fatty Liver Disease (MAFLD), a consensus-driven novel nomenclature for Non-Alcoholic Fatty Liver Disease (NAFLD), the most common chronic liver disease worldwide. Both increased ammonia production by gut microbiota and decreased ammonia hepatic removal due to impaired hepatic urea cycle activity or disrupted glutamine synthetase activity may contribute to hepatic ammonia accumulation underlying steatosis, which can eventually progress to hyperammonemia in more advanced stages of steatohepatitis and overt liver fibrosis. Furthermore, our group recently showed that augmented hepatic ammoniagenesis *via* increased glutaminase activity and overexpression of the high activity glutaminase 1 isoenzyme occurs in Fatty Liver Disease. Overall, the improved knowledge of disrupted nitrogen metabolism and metabolic miscommunication between the gut and the liver suggests that the reestablishment of altered gut-liver axis nitrogenous balance is an appealing and attractive therapeutic approach to tackle Fatty Liver Disease, a growing and unmet health problem.

## Introduction

### Ammonia, a central element in whole-body nitrogen metabolism

Ammonia is an inorganic nitrogen waste product metabolized and produced in all tissues and the principal culprit of hepatic encephalopathy (HE), a spectrum of neuropsychiatric abnormalities derived from liver dysfunction (1). Elevated blood ammonia, hyperammonemia, results from an imbalance between the ammonia produced and the body capacity to metabolize or remove it. In the human body under healthy conditions, ammonia is produced mostly in the gut by three main mechanisms: hydrolysis of urea by bacterial urease, bacterial protein deamination, and intestinal mucosal glutamine metabolism. Although the colon is conventionally assumed to be the major site of gut-ammonia production, recent evidence indicates that the stomach and small intestine are also involved (2). In the opposite, hepatic urea cycle is a well-described metabolic pathway for ammonia detoxification. Five urea cycle enzymes (UCEs) [carbamoyl phosphate synthetase I (CPS-1), Ornithine transcarbamoylase (OTC), argininosuccinate synthetase (ASS), argininosuccinate lyase (ASL) and arginase 1 (ARG1)] and 2 membranes transporters mediate the conversion of toxic ammonia into non-toxic urea that is excreted in the urine, ureagenesis (3). Hepatic ammonia metabolism is a highly zonated process being ureagenesis exclusively restricted to the periportal zone where the portal blood that carries ammonia from the gut first passes. Hepatic ammonia escaping ureagenesis is further excreted by means of the glutamine synthetase (GS) enzyme that catalyzes the ATP-dependent condensation of ammonia and glutamate to glutamine and solely expressed in the pericentral zone of the liver lobule. Failures in any of the gene coding for UCEs gives rise to a series of inherited life-threatening conditions overall characterized by aberrant ammonia accumulation, the urea cycle disorders (UCDs) (4). Also, the liver-specific GS-deficient mice present hyperammonemia and show increased locomotion, impaired fear memory, and a slightly reduced life span (5). By using liver specific and whole-body GS-KO mice together with stepwise increments of enterally or intravenously administered ammonium carbonate to challenge ammonia detoxification, Hakvoort and colleagues established that urea cycle and GS contribute equally to hepatic ammonia detoxification (6).

### Glutamine, a major nontoxic interorgan nitrogen carrier, and hepatic glutamine metabolism

**Figure 1:**
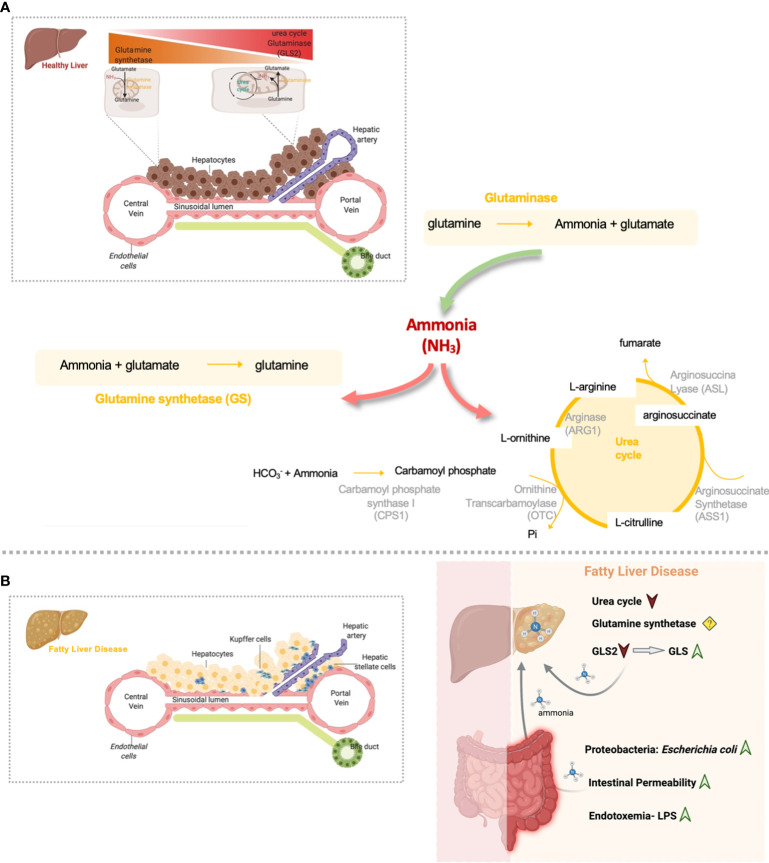
Overview of hepatic nitrogen metabolism homeostasis and disrupted gut-liver nitrogen metabolism in Fatty Liver Disease. **(A)** Both ammonia and glutamine hepatic metabolism are highly zonated processes. Hepatic ammonia enters in the liver from the gut where it can be eliminated by the urea cycle activity. In alternative, periportal hepatic glutaminase can convert glutamine to ammonia. Glutaminase and gut-derived ammonia that is not excreted in the periportal urea cycle, escapes to the pericentral region where is further eliminated by means of the glutamine synthetase (GS) enzyme. This enzyme converts ammonia back to glutamine and closing the glutamine cycle in order to maintain glutamine homeostasis. **(B)** Nitrogen metabolism homeostasis is disrupted in Fatty Liver Disease. Overall, inflamed gut in Fatty Liver Disease is characterized by the accumulation of proteobacteria Escherichia coli, very active in the production of ammonia. Gut-derived ammonia efflux through the portal vein can damage the liver directly or in alternative can exert detrimental effects on gut permeability and might indirectly contribute to NAFLD facilitating toxic molecules drainage into the portal blood. Additionally, it is possible that endotoxin and inflammation may contribute to increased uptake of ammonia from the gut into the bloodstream and thereby contribute to the latter’s toxic effect on the liver. In addition, Fatty liver Disease is characterized by diminished hepatic urea cycle activity and a switch from the low activity GLS2 to the high activity glutaminase, GLS, that together induce the accumulation of ammonia content in the liver. Ammonia can further drive fibrosis by promoting hepatic stellate cells activation, the main fibrogenic cell type. (Created by Biorender.com).

Glutamine, the most abundant amino acid in the body, much like ammonia is a major player in whole-body nitrogen metabolism. In the liver, glutamine metabolism is zonated, where glutamine is usually converted to glutamate and ammonia by the periportal mitochondrial enzyme glutaminase. Glutamate is further converted to α-ketoglutarate (αKG) by glutamate dehydrogenase (GDH) to enter the tricarboxylic acids (TCA) cycle. Ammonia produced during glutamine breakdown by glutaminase in periportal hepatocytes, together with ammonia derived for the gut entering liver through the portal vein, is partially removed by the urea cycle activity. Unremoved ammonia is delivered *via* the blood stream to pericentral hepatocytes for efficient hepatic ammonia detoxification through GS activity that converts ammonia into glutamine completing the intrahepatic cycling of glutamine (**Figure 1A**). *In vivo* experiments suggest that changes in net hepatic glutamine balance are mainly regulated by glutaminase activity, with the flux through glutamine synthetase being relatively constant (7). The glutaminase family consists of two isoenzymes, the kidney-type glutaminase (*GLS*, also known as *GLS1*) and the liver-type glutaminase (*GLS2*) genes. Healthy adult intestine and kidney express the *GLS* gene whereas the *GLS2* gene is highly expressed in the healthy adult liver (8).

## Metabolic dysfunction-Associated or Non-Alcoholic Fatty Liver Disease (MAFLD/NAFLD)

*Metabolic Dysfunction-Associated Fatty Liver Disease (MAFLD), a consensus driven novel nomenclature for Non-Alcoholic Fatty Liver Disease (NAFLD)* (9, 10)*, and from now on referred to as Fatty Liver Disease, comprehends a spectrum of conditions characterized by hepatic fat accumulation which can progress to inflammation, fibrosis and eventually leading to cirrhosis and hepatocellular carcinoma. Fatty Liver Disease is the most common type of chronic liver disease worldwide with an updated estimated prevalence of nearly 40%* (11)*, the leading cause of liver-related morbidity and mortality* (12)*, and often associated with obesity, insulin resistance, and diabetes* (13, 14).

### Ammonia in fatty liver disease

Disturbed interorgan trafficking of ammonia is a feature of Fatty Liver Disease. Indeed, our group and others showed that hepatic ammonia is increased in mouse models of diet-induced steatohepatitis as well as in Fatty Liver Disease patients’ liver biopsies (15–18). On the other hand, hyperammonemia, a distinguishable feature in advanced stages of cirrhosis and liver failure, was solely reported in mouse models of advanced fibrosis whereas it remains normal in animal models of steatosis and early stages of steatohepatitis (17, 19). In agreement, Felipo et al. described that patients with liver cirrhosis are hyperammonemic, Fatty Liver Disease patients have normal blood ammonia and patients with advanced steatohepatitis present mild hyperammonemia (20). Of interest, *in vivo* experiments in mouse models of early Fatty Liver Disease have shown that blood ammonia concentrations are significantly elevated in the portal vein blood (21), which suggests that a still functional hepatic ammonia extraction by the liver may reestablish normal blood ammonia levels.

### Gut-derived ammonia in fatty liver disease

Compelling evidence links the gut microbiome, intestinal barrier integrity, and the accumulation of fat in the liver. Indeed, dietary factors may alter the gut microbiota and intestinal barrier function, favoring the occurrence of metabolic endotoxemia and low-grade inflammation, contributing to the development of obesity and Fatty Liver Disease (revised in (22)). Gut-derived ammonia efflux through the portal vein can damage the liver directly or in alternative can exert detrimental effects on gut permeability and might indirectly contribute to Fatty Liver Disease facilitating toxic molecules drainage into the portal blood. Additionally, it is possible that endotoxin and inflammation, hallmarks of Fatty Liver Disease, may contribute to increased gut ammonia uptake into the bloodstream and accounting for the latter’s toxic effect on the liver (23). *In vitro* studies unraveled that the largest amount of ammonia is generated by gram-negative anaerobes, clostridia, enterobacteria such as *E. coli*, and *Bacillus spp* (24–26). Noteworthy, enterobacteria such *as E. Coli* are consistently enriched in steatosis and steatohepatitis (22). Shotgun metagenomic sequencing revealed a positive association between increased abundance of *E. coli* and advanced fibrosis in Fatty Liver Disease patients (27). Although these results suggest an accumulation of gut ammonia-producing bacteria in Fatty Liver Disease, the precise amount of ammonia generated by gut microbiota and its contribution to the accumulation of hepatic ammonia or hyperammonemia during steatohepatitis remains to be explored.

### Deregulated hepatic urea cycle activity in fatty liver disease

Ureagenesis through the urea hepatic cycle is impaired in animal models and patients with Fatty Liver Disease (16, 28, 29). Under these circumstances, reduced ureagenesis is related to decreased regulation of the UCEs at the gene level *via* hypermethylation of promoter regions of these genes (16, 30). However, reduction in the functional capacity for ureagenesis is more pronounced in simple steatosis than in steatohepatitis (29). Though post-transcriptional changes of the urea cycle genes cannot be ruled out, this difference is most probably a result of increased glucagon levels or inflammatory mediators, known to stimulate ureagenesis, that are augmented in steatohepatitis and not in simple steatosis. In the last years, steatosis-driven downregulation of urea cycle enzymes genes has been associated to the detrimental effects of long-chain fatty acids (31) or, in alternative, changes in the methylation index and methionine cycle rates observed in Fatty Liver Disease (32).

### Glutamine synthetase activity in fatty liver disease

Glutamine synthetase expression is decreased in cirrhosis independently of the etiology of the patients (33) and in drug-induced liver injury (34). In the opposite, GS is increased in regenerative states such as chronic hepatitis, focal nodular hyperplasia, peritumoral hyperplasia and some hepatocellular neoplasms (33). In pre-clinical mouse models of Fatty Liver Disease, GS protein expression was shown to be augmented (19, 21). Likewise, Eriksen et al. described that GS gene expression is augmented in patients both in simple steatosis and steatohepatitis compared to healthy liver lean and obese controls (29). In agreement, increased protein GS was also reported by our group in patients with early steatohepatitis (19). However, other studies have shown that hepatic protein GS expression decreased progressively in patients with steatosis and almost disappeared in steatohepatitis (16). Discrepancies reported in literature can reflect a lack of specificity of some antibodies used for the immunohistochemical staining or in alternative a mismatch between the regulation at the transcriptionally or post-transcriptional level of GS. Indeed, the GS gene is transcriptionally activated by glucocorticoid hormones in a tissue-specific fashion. However, at the ultimate level, the GS enzyme expression if governed by a post-transcriptional mechanism where GS protein turnover dependent of 26S proteosome is increased by a product feedback mechanism after glutamine stimulation (35). Further studies are necessary to better understand the spatiotemporal regulation of GS expression in the progression of Fatty Liver Disease.

### Glutamine and hepatic glutaminase activity in fatty liver disease

Glutamine metabolism homeostasis is disrupted in Fatty Liver Disease. Although serum glutamine is not significantly altered between healthy controls and patients with steatohepatitis, serum glutamate, a metabolite which metabolic pathways are closely related to glutamine, is augmented in patients with steatohepatitis (19). Similar findings were reported by Kuo et al., where serum glutamine remains constant as fibrosis severity worsens, but glutamate, the glutamate/glutamine ratio, the TCA cycle intermediate α-KG, and their downstream metabolites in the TCA cycle all increase as fibrosis severity worsened. Other study has shown that glutamine concentrations were significantly elevated in the hepatic vein and heart blood of Western diet-fed mice, a mouse model of steatohepatitis, but not in the portal vein blood (21). Differences in glutamine concentrations between the hepatic and portal veins are consistent with increased hepatic GS expression in Fatty Liver Disease (21).

Hepatic glutamine metabolism is mostly regulated by the actions of the glutaminase enzyme (7). Eriksen et al. described that *GLS2* expression, the liver type glutaminase, is decreased in steatohepatitis (29). In addition, recent findings from our group and others unraveled that the high-activity isoenzyme 1 of glutaminase (GLS) is overexpressed in mouse models and patients with steatohepatitis, which translates into an overall increase of hepatic glutaminase activity (19, 36) (**Figure 1B**). Importantly, the overexpression of GLS was detected in steatotic hepatocytes in pre-clinical and clinical samples of Fatty Liver Disease, with its transcriptional regulation still not fully understood (19). The relevance of glutamine metabolism in other types of hepatic cells during Fatty Liver Disease has also been addressed. Indeed, activation of HSCs, the main fibrogenic cell type, is highly dependent on glutaminolysis and GLS (36), suggesting that glutaminolysis is a potential diagnostic marker and therapeutic target during the progression from steatohepatitis to fibrosis (37). In addition, glutamine is one of the main sources of energy for Kupffer and endothelial cells (38), with GLS stimulating the proliferation, migration, and survival of the latter (39). However, to our knowledge the relevance of glutaminase alterations in Kupffer and endothelial cells underlying Fatty Liver Disease has not been specifically addressed.

## Modulation of nitrogen metabolism in fatty liver disease: Therapeutic approaches

Deregulated ammonia and glutamine homeostasis are hallmarks of Fatty Liver Disease. Therefore, is not surprising that therapeutic approaches modulating the gut-liver nitrogen metabolism are potentially relevant for the treatment of Fatty Liver Disease.

### Targeting gut microbiota in fatty Liver disease therapy

The gut is a potential source of systemic ammonia in Fatty Liver Disease; thus, capturing part of the gut ammonia may mitigate disease symptoms. Even though the beneficial effects of targeting ammonia-producing gut microbiota in advanced stages of chronic liver disease, such as cirrhosis, have been previously explored, studies addressing the effects of the specific targeting of ammonia-producing microbiota in Fatty Liver Disease are scarce.

Non-absorbable disaccharides, such as lactulose, are the first-line therapy for patients with hyperammonemia underlying UCDs and cirrhosis related HE (40–42). It has been shown that lactulose treatment ameliorated hepatic inflammation in animal models with steatohepatitis but could not completely prevent the development of steatohepatitis (43, 44). The antibiotic rifaximin, often used in combination with lactulose, has become the most effective antibiotic of choice in the treatment of hyperammonemia (45). Whereas some studies have shown that Rifaximin therapy appears to be effective and safe in modifying steatohepatitis through reduction of serum endotoxin and improvement of insulin resistance, proinflammatory cytokines, CK-18, and liver fat score (46, 47), other studies do not indicate a clear beneficial effect of rifaximin in patients with Fatty Liver Disease (48).

Probiotics can also reduce the total amount of ammonia in the portal blood by inhibiting bacterial urease activity. As most probiotics produce acids that reduce the pH in the intestine, ammonia absorption also decreases (49). In addition, probiotics reduce inflammation and oxidative stress in liver cells which further leads to increased hepatic clearance of ammonia (50–53). Even though several studies and clinical trials have encouraged the use of probiotic supplementation as promising and safe therapeutic approach in Fatty Liver Disease, nowadays the efficacy of probiotics in the management of these conditions remains limited to hypotheses.

Fecal microbiota transplantation (FMT) is an emerging treatment approach that is aimed at rebuilding intestinal microbiota to treat diseases and has been shown to attenuate hyperammonemia in HE animal models (54, 55). FMT may reduce ammonia synthesis by altering the gut microbiota composition to a taxon low in urease, diminish uptake of ammonia by reestablishing the integrity of the intestinal barrier and increase ammonia clearance by improving liver function. Shen et al. depleted animals of their preexisting gut microbiota and then inoculated with altered Schaedler flora (ASF), a defined consortium of 8 bacteria with minimal urease gene content. This protocol resulted in establishment of a persistent new community that promoted a long-term reduction in fecal urease activity and ammonia production. In a murine model of hepatic injury, ASF transplantation was associated with decreased morbidity and mortality (56). In another study, Kurtz et al. modified the oral probiotic *Escherichia coli nissle 1917* to create a strain (*SYNB1020*) that produces L-arginine and consumes ammonia. *SYNB1020* was shown to decrease systemic hyperammonemia in a mouse model of thioacetamide (TAA)-induced liver injury and phase I clinical trial showed a significant clinical effect and safety (57).

Finally, supplementation with Yaq-001, a non-absorbable synthetic carbon with high adsorptive capacity for bacterial products including LPS and pro-inflammatory cytokines, can reduce the transintestinal migration of gut microbiota and related metabolites, such as ammonia, bacteria-derived products, acetaldehyde, hydrophobic bile acids, and inflammation factors, including TNF-α and IL-6 in cirrhotic rats (58). This compound has also been shown to be safe and well tolerated in decompensated cirrhotic patients (59) and further studies in animal models and patients with Fatty liver Disease can provide insightful results.

### Therapeutic strategies to restore hepatic ammonia homeostasis either increasing ureagenesis or inhibiting glutaminase in fatty liver disease

Therapeutic strategies to reestablish ammonia content by diminishing urea cycle activity in patients with UCDs, cirrhosis and liver failure, have been developed in the last years. In fact, treatment with sodium benzoate to UCDs patients effectively decreases the blood ammonia level by reducing glycine metabolism in the liver, kidney, and brain. Other therapeutic option is sodium phenylacetate/phenylbutyrate that is rapidly oxidized to phenylacetate (PA) which conjugates to glutamine in the liver and is excreted as phenylacetylglutamine (PAGN) by the kidneys. In alternative, ornithine phenylacetate (OP) that on one hand uses PA to condensate to hepatic glutamine and excreted as PAGN, and on the other hand uses ornithine to boost urea cycle activity, effectively reduced ammonia levels in bile duct ligated rats, a mouse model of hepatic cholestasis, and in cirrhosis (60, 61). For the treatment of Fatty liver Disease, OP has been shown to reduce hyperammonemia whilst ameliorating steatohepatitis and fibrosis in mouse models (16, 17). In addition, hepatic ammonia content can be potentially rescued by inhibiting the activity of hepatic glutaminase. On this basis, our group and others have recently shown that both the specific silencing of hepatic GLS isoenzyme by using molecular approaches based on small interference RNA technology or its inhibition by using small chemical inhibitors can ameliorate steatohepatitis and fibrosis in pre-clinical mouse models of Fatty Liver Disease (19, 36). Finally, in the last years some *in vivo* studies have been carried out to address the effects of glutamine supplementation in the treatment of Fatty Liver Disease, with some of them showing a certain protective effect (62–64). **Table 1** summarizes the current therapeutic approaches that have been used to date for the modulation of nitrogen metabolism in Fatty Liver Disease.

**Table 1:** Current therapeutic approaches modulating nitrogen metabolism in pre-clinical and clinical studies in Fatty Liver Disease.

		Phase of the study	Main findings	Refs
**Modulation of gut microbiota**	Lactulose	Pre-clinical: Animal models	Improve hepatic inflammation Cannot prevent steatohepatitis development	(43, 44)
	Rifaximin	Clinical trials and observational studies	Safe and effective in modifying steatohepatitis No beneficial effect	(46–48)
	Fecal microbiota transplantation (FMT)	Pre-clinical: Animal models	Stools derived from healthy rodents could correct gut microbiota in steatohepatitis mice	(56)
	Engineering/FMT (SYNB1020)	Clinical trial: phase 1	Proven to be safe and effective in lowering ammonemia.	(57)
	Yaq-001	Clinical trial: programmed	A safety and efficacy study for the therapy of Fatty Liver Disease is being conducted	(58, 59)
**Targeting hepatic ureagenesis/glutaminase**	Ornithine phenylacetate (OP)	Pre-clinical: Animal models	Reduced hyperammonemia. Improved steatohepatitis and fibrosis	(16, 17)
	Glutaminase inhibitor: BPTES	Pre-clinical: Animal models	Reduced fibrosis in mouse models of CCl_4_-induced liver fibrosis	(36)
	GLS silencing	Pre-clinical: Animal models	Ameliorated steatohepatitis in mouse models of diet-induced steatohepatitis without significant fibrosis	(19)
**Glutamine supplementation**	Glutamine supplement in diet	Pre-clinical: Animal models	Glutamine has a certain protective effect in Fatty Liver Disease	(62–64)

## Concluding remarks

Fatty Liver Disease is characterized by increased hepatic ammonia content in early stages of steatosis followed by mild hyperammonemia in more advanced stages of steatohepatitis and fibrosis (15–18, 20). In addition, glutamine homeostasis is altered in Fatty Liver Disease as shown by increased serum glutamate/glutamine ratio in these patients (19, 36, 37). Fatty Liver Disease is characterized by gut dysbiosis and accumulation of the enterobacteria *E. coli*, very active in the production of ammonia (27). In addition, hepatic ammonia accumulation underlying Fatty Liver Disease is associated both with impaired liver ureagenesis through downregulation of the expression of UCEs (16, 28–30), and augmented hepatic glutaminase activity (19, 29, 36). Whether Fatty Liver Disease impairs nitrogen metabolism, or if these alterations in ammonia metabolism, ureagenesis ad glutaminase activity occur first and are the root of the appearance or progression of Fatty Liver Disease remains to be uncovered. However, the fact that UCDs patients seldom develop fatty liver as a consequence of disturbed ammonia metabolism (65), highlights the relevance of disturbed nitrogen metabolism as a potential driving mechanism in the onset of Fatty liver Disease.

Even though in the last years an effort has been carried out to define and understand the miscommunication between the gut and the liver in the deregulation of nitrogen metabolism in Fatty Liver Disease, there are still open questions. First, the fractional contribution of gut ammonia production, disturbed urea cycle function, glutamine synthetase expression or enhanced hepatic glutaminase activity contribution to altered ammonia and glutamine homeostasis in the context of Fatty Liver Disease remains to be addressed. We acknowledge that these pathways may contribute unequally and vary depending on several factors such as age, gender, body mass index, dietary regimen, stage of the disease and others. For example, the liver is a sexual dimorphic organ, and at least in healthy rats, significant sex-related changes in urea cycle were found (66). Therefore, studies addressing the discovery of organ-specific biomarkers panels to be used as surrogates of the main metabolic pathways underlying deregulated nitrogen metabolism in Fatty Liver Disease are relevant. These biomarkers will allow the classification and grouping in sub-types of patients presenting similar characteristics with respect to their gut-liver nitrogen metabolism. Secondly, the term MAFLD was coined, among other things, to avoid diagnosis exclusion based on alcohol consumption (9). Alcohol consumption is clearly under-reported in NAFLD (67) which precludes that many of the clinical studies presenting impaired nitrogen metabolism in the context of NAFLD may also include patients undergoing alcohol consumption. In agreement, earlier studies suggest that patients with acute and chronic alcoholic liver disease present hyperammonemia and decreased capacity for urea synthesis (68, 69). Thereby, it would be interesting to understand how alcohol modulates the gut-liver nitrogen metabolism independent of other dietary factors which can be successfully performed in *in vivo* mouse models. Also, although the gut and the liver are central in the regulation of whole-body nitrogen metabolism, other organs cannot be obliviated. In fact, Elfeki and Singal recently proposed that chronic kidney disease may contribute to the higher levels of ammonia among patients with Fatty Liver Disease (70). And finally, is crucial to find novel targets and more effective pharmacological agents or therapeutical approaches to modulate nitrogen metabolism.

In summary, the liver-gut axis nitrogen metabolism is disturbed in Fatty Liver Disease with significant changes in ammonia and glutamine homeostasis and this has led to the hypothesis that therapeutic strategies that modulate nitrogen metabolism can potentially be used for the clinical managements of Fatty Liver Disease.

## Author contributions

Writing and correction of the manuscript: TCD, JdH and MLM-C. All authors contributed to the article and approved the submitted version.
